# Akreos Adapt AO Intraocular lens opacification after vitrectomy in a diabetic patient: a case report and review of the literature

**DOI:** 10.1186/s12886-016-0268-3

**Published:** 2016-06-08

**Authors:** Dan Cao, Hongyang Zhang, Cheng Yang, Liang Zhang

**Affiliations:** Department of Ophthalmology, Guangdong General Hospital, Guangdong Academy of Medical Sciences, Guangzhou, China

**Keywords:** Opacification, Calcification, Hydrophilic acrylic intraocular lens, Diabetes

## Abstract

**Background:**

Postoperative optic opacification of hydrophilic acrylic intraocular lenses (IOLs) is an uncommon complication leading to IOL explantation. In the past decade, several studies reported that the granular deposits responsible for the opacification were probably calcium and phosphate salts; however, the exact mechanism causing calcification of IOLs is unknown. The aim of this study is to describe clinical and laboratory findings of a case of late postoperative opacification of an aspheric hydrophilic acrylic IOL (Akreos Adapt AO) after vitrectomy.

**Case presentation:**

A 60-year-old woman diagnosed with cataract and severe nonproliferative diabetic retinopathy (NPDR) underwent uneventful phacoemulsification and hydrophilic acrylic IOL (Akreos Adapt AO, Bausch & Lomb) implantation in both eyes. Seven months later, the woman came back with a complaint of blurry vision in the left eye. Fundus examination revealed vitreous hemorrhage in the left eye veiling the retinal detail. A 23-gauge vitrectomy with endolaser treatment was performed in the left eye. Ten months after the vitrectomy, the patient complained of decreased visual acuity in the left eye again. On slit-lamp examination, we observed a well circumscribed centrally and paracentrally located opacification within the pupillary area localized to the anterior surface of the IOL. The IOL was explanted from the left eye together with the capsular bag, and an iris-claw lens (Artisan Aphakia OPHTEC) was implanted. The explanted IOL was examined under pathological evaluation (alizarin red method).

**Conclusions:**

IOL opacification is a rare event. We described a case of postoperative opacification of the Akreos Adapt AO IOL after vitrectomy in a patient with proliferative diabetic retinopathy and found the deposits on the anterior surface of the IOL consisted of calcium aggregates. Given the higher frequency of postoperative opacification observed in diabetic patients, hydrophilic acrylic IOLs should be used with caution in patients with diabetes.

## Background

Postoperative optic opacification of hydrophilic acrylic intraocular lenses (IOLs) is an uncommon complication leading to IOL explantation. In the past decade, several studies reported that the granular deposits responsible for the opacification were probably calcium and phosphate salts [1–4]; however, the exact mechanism causing calcification of IOLs is unknown. The aim of this study is to describe clinical and laboratory findings of a case of late postoperative opacification of an aspheric hydrophilic acrylic IOL (Akreos Adapt AO) after vitrectomy.

## Case presentation

In February 2014, a 60-year-old woman with type 2 diabetes was referred to our hospital. She was diagnosed with cataract and severe nonproliferative diabetic retinopathy (NPDR) in both eyes. On examination she had best corrected visual acuity (BCVA) 0.02 in the right eye and 0.01 in the left eye. She underwent uneventful phacoemulsification and hydrophilic acrylic IOL (Akreos Adapt AO, Bausch & Lomb) implantation in both eyes. Two weeks after cataract surgery the BCVA in the left eye improved to 0.4. Then she had fundus fluorescein angiography (FFA) and received panretinal photocoagulation in both eyes.

In September 2014, the woman came back with complaint of blurry vision in the left eye. Fundus examination revealed vitreous hemorrhage in the left eye veiling the retinal detail. We performed a 23-gauge vitrectomy with endolaser treatment in the left eye.

**Fig. 1:**
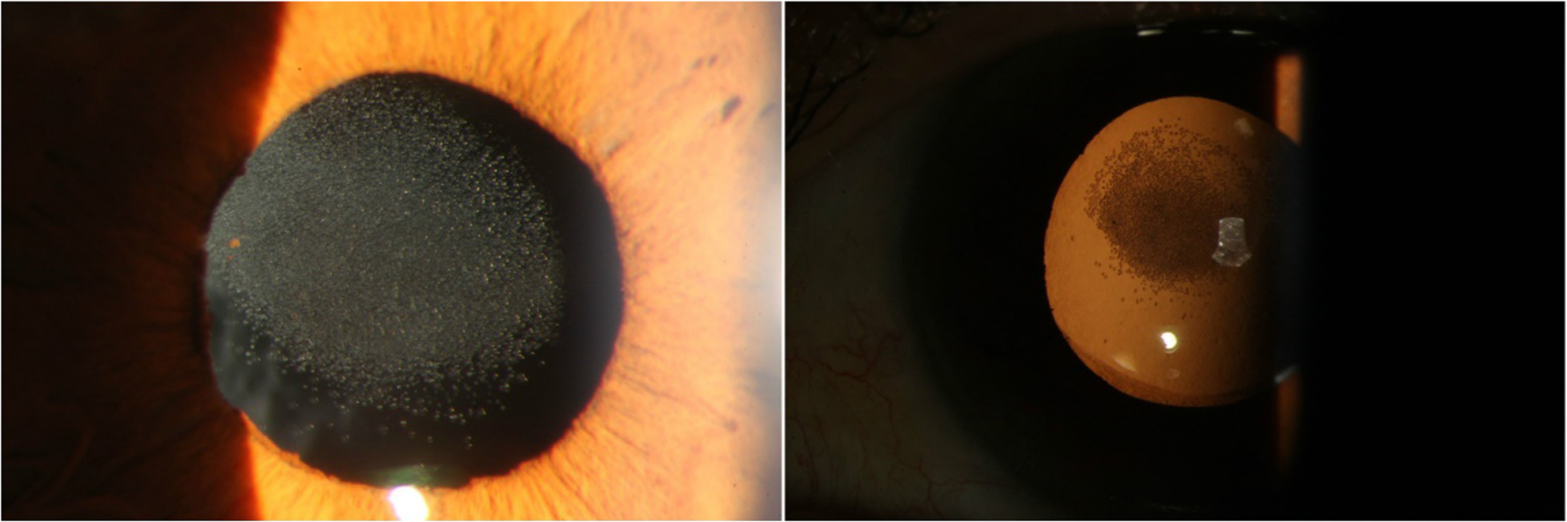
Slit-lamp photographs taken before IOL explantation

**Fig. 2:**
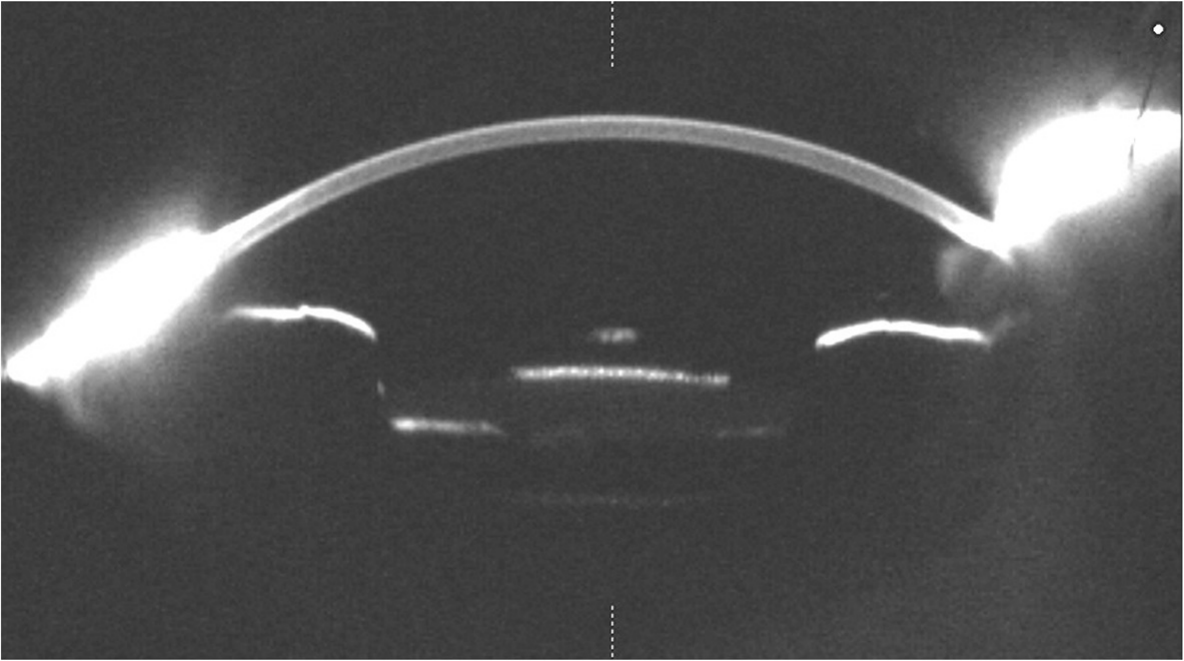
Light scatter was high at the anterior surface of the explanted IOL under Scheimpflug photography, within the area corresponding to the granular deposits

Ten months after the vitrectomy (July 2015), the patient complained of decreased visual acuity in the left eye again (the BCVA was 0.1). On slit-lamp examination, we observed a well circumscribed centrally and paracentrally located opacification within the pupillary axis localized to the anterior surface of the IOL (Fig. 1). Scheimpflug pictures taken by Pentacam (Oculus) showed increased light scatter on the IOL’s anterior surface (Fig. 2).

**Fig. 3:**
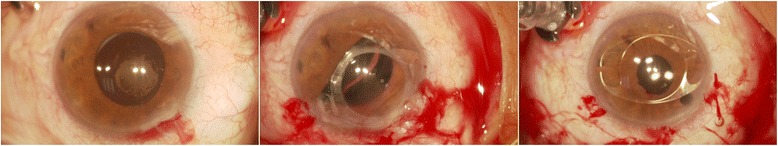
Surgical exchange of the opacified Akreos Adapt AO IOL. Opacified IOL explantation together with the capsular bag. An iris-claw lens was implanted

The IOL was explanted from the left eye together with the capsular bag, and an iris-claw lens (Artisan Aphakia OPHTEC) was implanted (Fig. 3). Three days after the operation, the BCVA improved to 0.2.

The explanted IOL was sent to research center of Guangdong Academy of Medical Sciences. The unstained IOL was evaluated and photographed under a light microscope (Olympus Optical Co.,Ltd.). Then the IOL was rinsed in distilled water, immersed in 1.0 % alizarin red solution (a special stain for calcium) for 10 min, rinsed again in distilled water, and reexamined under the light microscope.

**Fig. 4:**
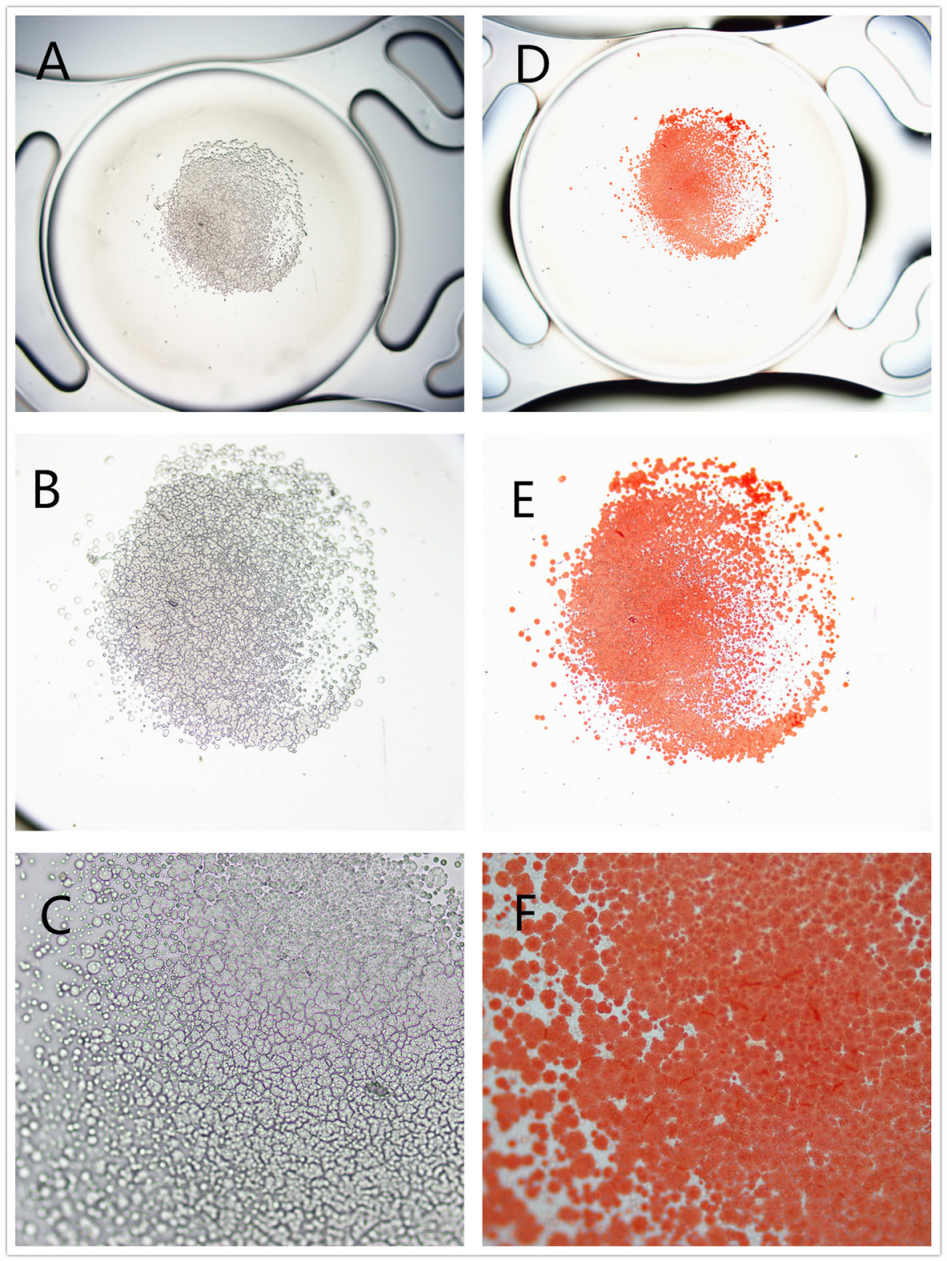
Light photomicrographs of the explanted IOL. Unstained photomicrographs showing the deposits on the anterior surface of the explanted IOL (**a**, original magnification × 20; **b**, ×100; **c**, ×200). The deposits stained positive with alizarin red. (**d**, original magnification × 20; **e**, ×100; **f**, ×200)

Light microscopy showed the presence of granular deposits distributed in an overall round pattern on the anterior surface of the IOL. The granules were stained positive for calcium (alizarin red method) (Fig. 4).

## Discussion

To date, postoperative opacification of modern hydrophilic acrylic IOLs has been reported in many cases. The five major hydrophilic acrylic IOLs include Hydroview (Bausch & Lomb), the SC60B-OUV (Medical Developmental Research, Inc.), ACRL-60 (Ophthalmed Inc.), MemoryLens (Ciba Vision) and AquaSense (Ophthalmic Innovations International, Inc.) [1–5]. Histopathological analysis and molecular surface analysis have been performed on the explanted opacified IOLs, and calcium and phosphate precipitations were found on the surface/subsurface and/or within the IOLs.

Akreos adapt AO is a modern aberration-free aspheric hydrophilic acrylic lens. Only sporadic cases of optic opacification involving the Akreos adapt AO IOL have been described (Table 1). In 2008, Shiu Ting Mak et al. [6] reported the first case of opacification of the Akreos Adapt AO IOL. The explanted IOL in their case was examined under scanning electron microscopy, and foci of calcium and phosphorous were seen in the IOL material. Liliana Werner et al. [7] described another two cases of localized opacification of Akreos adapt AO IOL after procedures using intracameral injection of air or gas. It was theorized that a metabolic change in the anterior chamber due to the presence of exogenous gas in the eye, or an exacerbated inflammatory reaction after multiple surgical procedures might cause the calcification of IOL. Later Mattro Forlini et al. [8] and Chong Eun Lee et al. [5] each outlined a single case developing optic opacification after glaucoma surgeries using Akreos adapt AO IOL separately; however, pathologic analysis were unavailable in those two cases.

**Table 1:** Six cases of opacifiaction of Akreos Adapt AO IOL reported so far

Reporter	Associated ocular conditions	Other history	Other ocular surgeries/procedures
Shiu Ting Mak et al.	a history of anterior uveitis	ischemic heart disease, hypertension, and gout	
Liliana Werner et al.	Fuchs dystrophy		Descemet-stripping automated endothelial keratoplasty (DSAEK)
Liliana Werner et al.	Fuchs dystrophy	diabetes	repeated DSAEK with complete gas fill
Mattro Forlini et al.	glaucoma	diabetes and hypertension	Ex-press device implantation
Chong Eun Lee et al.	neovascular glaucoma	diabetes	Ahmed valve implantation
current study	PDR	diabetes	23-gauge vitrectomy

In the present study, the deposits on the explanted IOL stained positive with alizarin red (a special stain for calcium). The patient had a history of type 2 diabetes for more than five years. She received phacoemulsification and was implanted with Akreos adapt AO IOLs in both eyes; however, only the left eye which presented with vitreous hemorrhage and received vitrectomy developed calcification of the IOL. We presume that preexisting diabetic retinopathy, inflammatory reaction after vitrectomy or a breakdown of the blood-aqueous barrier (BAB) may be responsible for the opacification.

We noticed a higher rate of diabetes in patients with opacification of Akreos Adapt AO IOLs (four out of six patients having concomitant diabetes). Previous studies also supported that IOL opacification was most common in patients with systemic diseases such as diabetes [9, 10]. First of all, in cases of diabetic retinopathy (DR), where many pathological conditions such as ischemia/hypoxia, shear stress and inflammation play a role, intravitreal levels of adenosine triphosphate (ATP) are significantly increased as compared with those in non-diabetic controls [11]. Therefore, increased calcium influx is evoked by intravitreal ATP. Secondly, in the eyes of DR a higher concentration of intravitreal protein is identified. This is involved in the production of angiotensin I and elevates the concentration of serum calcium. A combination of the two hypotheses may lead to the higher incidence of IOL calcification in diabetic patients. However, we are unable to establish a correlation between these complications and diabetes. Further study is warranted to continue monitoring cases of hydrophilic acrylic IOL calcification to verify the percentage of cases associated with diabetes or DR.

## Conclusions

IOL opacification is a rare event. We described a case of postoperative opacification of Akreos Adapt AO IOL after vitrectomy in a patient with proliferative diabetic retinopathy and found the deposits on the anterior surface of the IOL consisted of calcium aggregates. Given the higher frequency of postoperative opacification observed in diabetic patients, hydrophilic acrylic IOLs should be used with caution in patients with diabetes.

## Abbreviations

ATP, Adenosine triphosphate; BCVA, Best corrected visual acuity; DR, Diabetic retinopathy; DSAEK, Descemet-stripping automated endothelial keratoplasty; FFA, fundus fluorescein angiography; IOL, Intraocular lens
